# A Matter of Balance: Motor Control is Related to Children’s Spatial and Proportional Reasoning Skills

**DOI:** 10.3389/fpsyg.2015.02049

**Published:** 2016-01-12

**Authors:** Andrea Frick, Wenke Möhring

**Affiliations:** ^1^Department of Psychology, University of BernBern, Switzerland; ^2^Department of Psychology, University of FribourgFribourg, Switzerland; ^3^Department of Psychology, Temple UniversityPhiladelphia, PA, USA

**Keywords:** cognitive development, motor control, balance, proportional reasoning, spatial scaling, inhibitory control, working memory, executive functions

## Abstract

Recent research has shown close links between spatial and mathematical thinking and between spatial abilities and motor skills. However, longitudinal research examining the relations between motor, spatial, *and* mathematical skills is rare, and the nature of these relations remains unclear. The present study thus investigated the relation between children’s motor control and their spatial and proportional reasoning. We measured 6-year-olds’ spatial scaling (i.e., the ability to reason about different-sized spaces), their mental transformation skills, and their ability to balance on one leg as an index for motor control. One year later (*N* = 126), we tested the same children’s understanding of proportions. We also assessed several control variables (verbal IQ and socio-economic status) as well as inhibitory control, visuo-spatial and verbal working memory. Stepwise hierarchical regressions showed that, after accounting for effects of control variables, children’s balance skills significantly increased the explained variance in their spatial performance and proportional reasoning. Our results suggest specific relations between balance skills and spatial as well as proportional reasoning skills that cannot be explained by general differences in executive functioning or intelligence.

## Introduction

The idea that cognitive and motor development are closely intertwined goes back to early developmental theories (e.g., Gesell and Thompson, 1934; Piaget, 1952). For example, Piaget claimed that the emergence of cognitive skills is based on sensorimotor experience. With increasing motor activity, infants become equipped with new possibilities to discover their environment and understand themselves as active agents, resulting in increasingly differentiated cognitive structures. These early seminal theories laid the basis for later theoretical frameworks (e.g., Gibson, 1988; Bushnell and Boudreau, 1993; Diamond, 2000) and inspired many studies supporting a close relation between motor and cognitive development in general and spatial cognition in particular. Another line of research showed a specific connection between performance on spatial cognitive tasks and mathematical thinking (for a review, see Mix and Cheng, 2012). However, research examining the relations between motor, spatial, *and* mathematical skills is rare, and the nature of these relations remains unclear. The present study thus investigated the relation between children’s balance skills and their spatial scaling, mental transformation, as well as proportional reasoning skills, using a longitudinal approach.

### Relation Between Motor and Cognitive (Spatial) Skills

Previous research has shown that providing infants with increased experience in a particular motor skill such as reaching enhanced their object segregation skills (Needham, 2000), visual exploration of faces and objects (Libertus and Needham, 2010, 2011), and understanding of intentional movements (Sommerville et al., 2005; for a review, see Hauf, 2007). It has also been shown that the onset of motor milestones such as independent sitting or walking had beneficial effects on infants’ perceptual abilities (Campos et al., 1992; Soska et al., 2010) and on their social-emotional development (for a review, see Campos et al., 2000). Moreover, longitudinal studies indicated that early gross-motor skills predicted later cognitive performance, such as executive functions or perceptual processing (Murray et al., 2006; Piek et al., 2008). Even at old age, motor abilities have been found to be associated with cognitive performance (e.g., perceptual speed and executive control, Voelcker-Rehage et al., 2010).

Further evidence for a close link between motor development and cognitive performance comes from a study by Davis et al. (2011). Using standardized tests, the authors found that cognitive and motor skills were closely related in children between 4 and 11 years of age. Interestingly, a principal component analysis revealed that children’s visual processing and fine manual control showed considerable cross-loadings on both a cognitive and a motor factor. The authors concluded that the interrelation between cognitive and motor development may be underpinned by these specific skills. However, both of these variables were assessed using tasks that required high-level spatial processing. For example, visual processing was assessed by having participants construct a copy of an abstract design using 3D shapes or find the fastest route across a grid with obstacles. Fine manual control tasks included coloring, drawing, folding, cutting-out forms, and copying geometric shapes. Therefore, it could be argued that spatial skills were in fact responsible for the observed correlations.

Indeed, many studies found specific relations between motor ability and spatial cognition, with a particular focus on mental rotation. They showed that manual experience enhanced infants’ mental rotation performance (e.g., Möhring and Frick, 2013; Schwarzer et al., 2013b; Frick and Wang, 2014), and that infants’ locomotor experience was associated with a better understanding of rotational movements (Frick and Möhring, 2013; Schwarzer et al., 2013a). Furthermore, correlational evidence indicated that 5- to 6-year-olds’ motor control and coordination skills were associated with mental rotation (Jansen and Heil, 2010). More specifically, Jansen and colleagues (Jansen et al., 2011; Jansen and Kaltner, 2014), showed that balance skills were especially important in predicting 10-year-olds’ and older adults’ mental rotation performance.

### Relation Between Spatial and Mathematical Skills

Another body of research has provided mounting evidence suggesting that spatial reasoning is closely related to mathematical understanding (for a review, see Mix and Cheng, 2012). For example, findings have demonstrated connections between mental object rotation and arithmetic skills in adolescents (Reuhkala, 2001; Kyttälä and Lehto, 2008), as well as mental rotation and performance on the math subtest of the Scholastic Aptitude Test (Casey et al., 1995). Longitudinal studies indicated that children’s mental transformation skills predicted their accuracy in locating numbers on a line, which in turn was related to their later mathematical proficiency (Gunderson et al., 2012; LeFevre et al., 2013). Furthermore, a training study showed that spatial-numerical training was more beneficial for preschoolers’ mental number line performance and mathematical achievement than purely numerical training (Fischer et al., 2011). Considering this evidence for a strong link between spatial and mathematical skills, as well as the literature showing a close connection between spatial abilities and motor processes outlined above, the question emerges whether motor skills are also related to children’s mathematical performance.

### Relation Between Mathematical and Motor Skills

There actually is some evidence that motor skills may be associated with math performance. For example, Lopes et al. (2013) assessed motor coordination and academic achievement (Language and Math National Exams) in Portuguese 9- to 12-year-olds, and found that children with motor coordination deficits exhibited a higher probability of low academic achievement compared to children with normal motor coordination. This result is all the more alarming considering that 52% of the children were found to exhibit motor coordination deficits and no child showed good motor coordination. Other research (Carlson et al., 2008) found that the number of hours of physical education per week was associated with higher mathematics performance in female (but not male) kindergarteners and first-graders. Furthermore, longitudinal findings (Luo et al., 2007) indicated that fine-motor skills significantly predicted mathematics achievement over time (kindergarten through first grade), and likely explained performance differences between ethnic groups in the United States.

However, many of the previous studies investigating a link between motor skills and academic achievement did not control for general cognitive abilities, which makes the results hard to interpret. For example, it is possible that the correlations were due the children’s general developmental status, and that some children were more mature and advanced in all of the variables measured. Such a scenario is not unlikely, as deficits in cognitive and motor development often co-occur (e.g., Piek et al., 2004) and motor skills are considered an important index for brain maturation (Magill, 1996). Another possibility is that a positive association between motor and cognitive skills is due to a specific relation. Consequently, in order to find out more about the specific nature of the relation between motor and cognitive abilities and possible shared processes, it is necessary to control for such general cognitive variables.

### Rationale and Aims of the Study

In the present study, we investigated whether motor control is related to 6-year-old children’s spatial skills as well as predictive for their understanding of proportions, using a longitudinal design. In contrast to previous studies, we controlled for general cognitive abilities as well as other possible covariates that will be described below. We tested children shortly before and after they transitioned to primary school, because spatial skills can be measured reliably but still develop considerably at this age, and would therefore exhibit large individual variance. Furthermore, we intended to assess basic mathematical skills (proportional reasoning) at an age when it was unlikely that children had received much formal educational input on the specific topic yet.

As an index for motor control, we assessed children’s ability to stand on one leg. Balance skills have previously been associated with cognitive ability in 5- to 6-year-old boys (Planinsec, 2002), reading and math skills in 7- to 11-year-olds (Knight and Rizzuto, 1993), spatial skills (Jansen et al., 2011; Jansen and Kaltner, 2014), and executive functioning in adolescents (Rigoli et al., 2012). Furthermore, balance is a prerequisite for many more complex motor skills (such as walking or riding a bicycle). Thus, it is relevant for locomotor abilities, which in turn are related to spatial skills early in life (Frick and Möhring, 2013; Schwarzer et al., 2013a). Yet, unlike many other motor tasks, balance does not require high-level spatial processing, and can therefore be considered a pure measure of motor control.

As measures of spatial skills, we assessed children’s mental transformation abilities, using the *Children Mental Transformation Task* (Levine et al., 1999). This test assesses children’s ability to mentally combine two shapes (by translation or rotation). Based on previous findings (Jansen et al., 2011), we expected that children’s mental transformation skills would be related to motor control. However, in extension to previous research that mainly focused on mental rotation, we also assessed children’s spatial scaling abilities using the *Spatial Scaling Test* (Frick and Newcombe, 2012). Spatial scaling refers to the ability to compare different-sized spaces. We expected to find a similar link to motor control, based on recent findings suggesting that spatial scaling is based on similar mental transformation strategies (transforming one space in *size* to match the other) as mental rotation (transforming one object in *orientation* to match the other; Möhring et al., 2014). The relation between spatial scaling and motor abilities has not been investigated to date. This is surprising given that spatial scaling is a foundational skill for understanding and using scaled representations (i.e., maps or models) and plays an important role for many daily and professional activities, such as using a map to navigate or a blueprint to build a skyscraper.

Scaling also has important educational implications and has been defined as an important and overarching theme for science education by the U.S. National Research Council (2012). In fact, previous research has indicated that children’s spatial scaling abilities are closely related to the ability to reason about proportions (Boyer and Levine, 2012; Möhring et al., 2015a). Proportional reasoning, in turn, has been associated with children’s formal fraction knowledge (Möhring et al., 2015b), raising the possibility that proportional reasoning might be an important precondition for children’s understanding of crucial mathematical concepts, such as fractions or divisions. Given these close relations, in the present study we tested the same children’s understanding of proportions one year later. Children were given the *Proportional Reasoning Task* (adapted from Möhring et al., 2015a), in which they were asked to rate how much cherry flavor would be tasted in different juice-water mixtures.

We also assessed several possible covariates. Previous studies have revealed that keeping postural control requires high-level cognitive processes such as attention (for a review, see Woollacott and Shumway-Cook, 2002). Motor control was also associated with inhibition and working memory (WM; Piek et al., 2004; Roebers and Kauer, 2009), with visuo-spatial WM being more important than verbal WM (Alloway and Temple, 2007; Rigoli et al., 2012). Moreover, WM was found to be related to mathematical performance in adults, typically developing children, and in children with math difficulties (for a review, see Raghubar et al., 2010). Similar to findings for motor control, visuo-spatial WM in particular was related to mathematical understanding (Kyttälä et al., 2003; Bull et al., 2008), and locating relational quantities on a number line (Vukovic et al., 2014).

Consequently, one potential factor underlying the motor-cognition link might be found in children’s executive functions. Indeed, Roebers et al. (2013) found that after accounting for executive functions (inhibition, cognitive flexibility, verbal WM), fine-motor skills no longer predicted children’s school achievement. Similarly, Lehmann et al. (2014) showed that after controlling for WM, balance skill was no longer related to children’s mental rotation performance. Thus, these studies point to a major contribution of executive functions to the motor-cognition link, which is why we included measures of children’s inhibitory control, verbal, and visuo-spatial WM. Finally, to control for general effects of intelligence, we also included a measure of verbal IQ (i.e., an IQ measure that is minimally related to spatial, mathematical, and motor skills). Furthermore, socio-economic status (SES) was assessed to control for general effects of children’s social environment.

## Materials and Methods

### Participants

The present research was conducted as part of a larger longitudinal study investigating how spatial skills in kindergarten are related to later school achievement. Children were recruited during their last kindergarten year in 24 different rural and urban kindergartens in Switzerland. Signed parental consent forms and children’s verbal assents were obtained prior to the study from 140 children (62 girls, mean age = 6.49 years, *SD* = 0.27, range = 6.01–6.99; 78 boys, mean age = 6.46, *SD* = 0.34, range = 5.99–7.01). One year later, the same children were tested again, except for 14 children, who had moved to a different school district (2), were sick on the day of assessment (1), or no longer had parental consent (11). The final sample for which both complete data sets were available comprised 126 children (55 girls, mean age = 7.55 years, *SD* = 0.28, range = 6.98–8.09; 71 boys, mean age = 7.53, *SD* = 0.35, range = 6.95–8.13). Procedures followed ethical guidelines and were approved by the Institutional Review Board of the University of Bern.

### Procedure

The first assessment (T1) was administered at the end of children’s last kindergarten year, before children transitioned to primary school. The second assessment (T2) took place at the end of first grade. T1 consisted of two test sessions, each lasting about 30 min, with about 1–2 weeks in between (*M* = 10.4 days; *SD* = 8.5 days). Children were tested individually in a separate room in their kindergarten. For most tests, materials were presented on a table, with the experimenter sitting orthogonally to the side of the participants. Children completed the *Spatial Scaling Test* (SST: Frick and Newcombe, 2012), and the *Children’s Mental Transformation Task* (CMTT, Levine et al., 1999) among four other tasks examining spatial transformation abilities^1^. Children also completed some tasks that assessed non-spatial skills, such as inhibitory control, verbal IQ, and balance. Furthermore, SES was assessed via parent questionnaires. At T2, visuo-spatial and verbal working memory were assessed along with proportional reasoning skills in one single session. In the following, the tasks that were at the focus of the present paper will be described in more detail; descriptions of the tasks that were not central to the present research question can be found in the respective publications (see Footnote 1). After each session, children were praised regardless of the level of their performance and received a small snack or toy.

### Measures

Children’s *balance* was measured as an index of gross-motor skills. Children were asked to stand on one leg as long as they could, while the experimenter measured the time (in s) using a stopwatch. If a child was only able to balance for a few seconds, he or she was instructed to relax, take a deep breath, and then allowed a second try. Hopping was not allowed and the test was ended after a maximum of 100 s.

**FIGURE 1:**
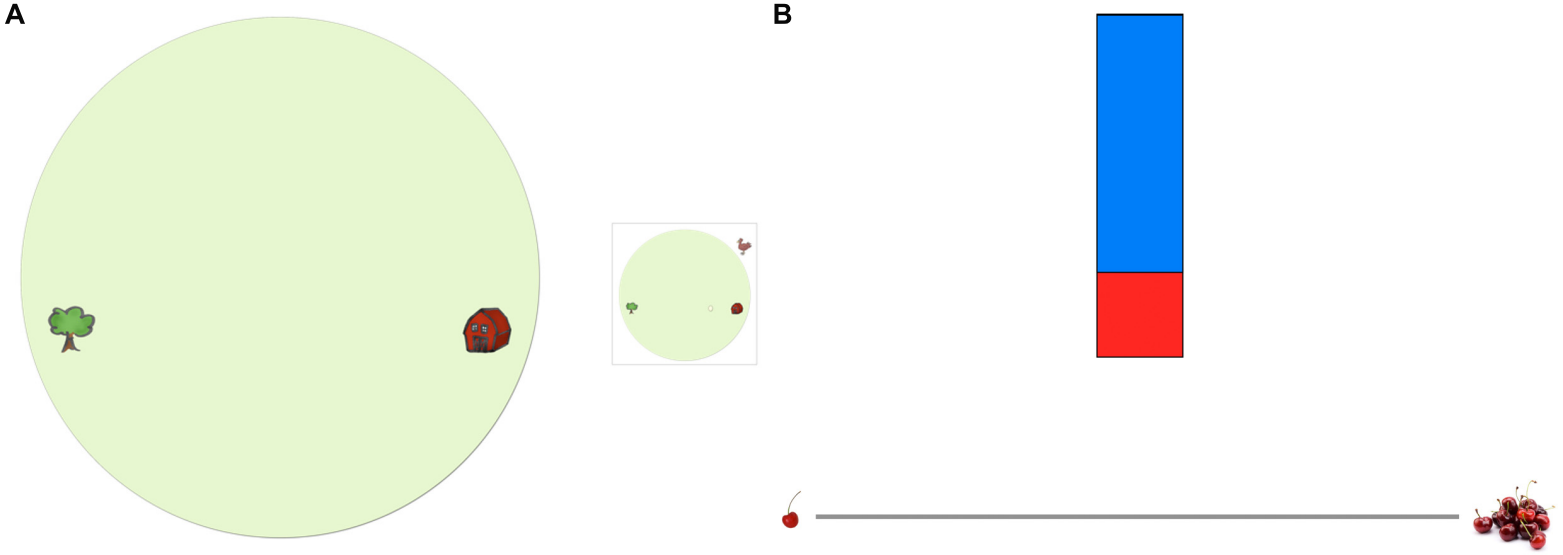
**(A)** Example for an item of the *Spatial Scaling Test* (circular field, scaling factor 1:4), and **(B)** example for an item of the *Proportional Reasoning Test* (two units of cherry juice vs. six units of water).

In the *Spatial Scaling Test* (SST, Frick and Newcombe, 2012), children were told a story about a farmer, whose chickens hid their eggs in the fields. They were presented with drawings of green “fields” (see **Figure 1A**). The shapes of the fields were rectangular (22 cm by 14 cm), long narrow strips (26 cm by 4 cm), or circular (20 cm in diameter; with two landmarks). In each trial, a map was placed directly to the right of the field. The map showed the same picture with a target object (egg) in it, and either had the same size or was smaller than the field, such that every distance on the map corresponded to a four times larger distance in the field. Children were asked to help the farmer find the eggs by placing a small rubber peg on the field in the same position where the picture (map) showed the egg. Every combination of scaling factor and field was presented four times, using different target locations, amounting to a total of 24 trials, which took approximately 7–8 min to complete. The experimenter marked the position and scored the responses after the experiment, using a transparency that showed concentric circles of increasing radii (1, 1.5, and 2 cm) around the target locations. Responses within these circles were scored with 1, 2/3, or 1/3 point, respectively, and summed across trials.

The *Children’s Mental Transformation Task* (CMTT) was adapted from Levine et al. (1999). Children were presented with two black shapes on white paper. They were asked to imagine what kind of shape the two pieces would form if moved together and choose among four presented alternatives. We used an abbreviated version of the original test, presenting 12 items in which the two pieces had to be translated horizontally and rotated 60° each to form the target shape, and 12 items that required a diagonal translation but no rotation. These 24 items took children approximately 6–9 min to complete.

The *Proportional Reasoning Task* was adapted from Möhring et al. (2015a). Children were told a story about a bear, who likes to drink cherry juice mixed with water. Then, children were presented with combinations of red and blue rectangles representing cherry juice and water that were 2 cm wide and of varying lengths (see **Figure 1B**). Children were asked to estimate the cherry taste of each mixture by drawing a mark on a horizontal line (15 cm). A single cherry to the left of the rating scale indicated a weak cherry taste; a heap of cherries to the right of the rating scale indicated a strong cherry taste. Two instruction trials presented mixtures that were not used in later test trials (first trial: 1 unit juice vs. 10 units water, second trial: 3 units juice vs. 0.3 unit water), and children were given corrective feedback. Children did not receive feedback on subsequent test trials, in which three levels of juice (2, 4, 6 units) and water (3, 6, 9 units) were combined in a full factorial design. These nine combinations were shown twice, amounting to 18 trials that were presented in a quasi-random order that avoided direct repetitions of factor levels. The task took approximately 5–6 min. After the task, the experimenter measured the locations of the marks on the rating scale (in mm).

*Inhibitory control* was measured using the *Fruit Stroop* task (Archibald and Kerns, 1999), in an adapted version by Röthlisberger et al. (2010). This task is appropriate for preschool or kindergarten children, because as opposed to the classic Stroop task, it does not require reading skills (MacLeod, 1991). Children saw a total of four A4 pages. The first page contained 25 colored squares (blue, yellow, red, green) and children were asked to name the colors of the squares, going through them row by row (baseline). The second page showed 25 colored fruits and vegetables, and children were again asked to name their colors (congruent). Page 3 showed the same fruits and vegetables in black-and-white (neutral), and page 4 in wrong colors (incongruent), and children were asked to name the colors they *should* have (e.g., a banana was shown in blue with the correct response being “yellow”). The task took approximately 6–7 min. An interference score was calculated after a formula suggested by Archibald and Kerns (1999), which calculates costs in response times when seeing incongruent colors, taking into account children’s baseline naming speeds. Higher scores indicate stronger interference (and lower inhibitory control).

**FIGURE 2:**
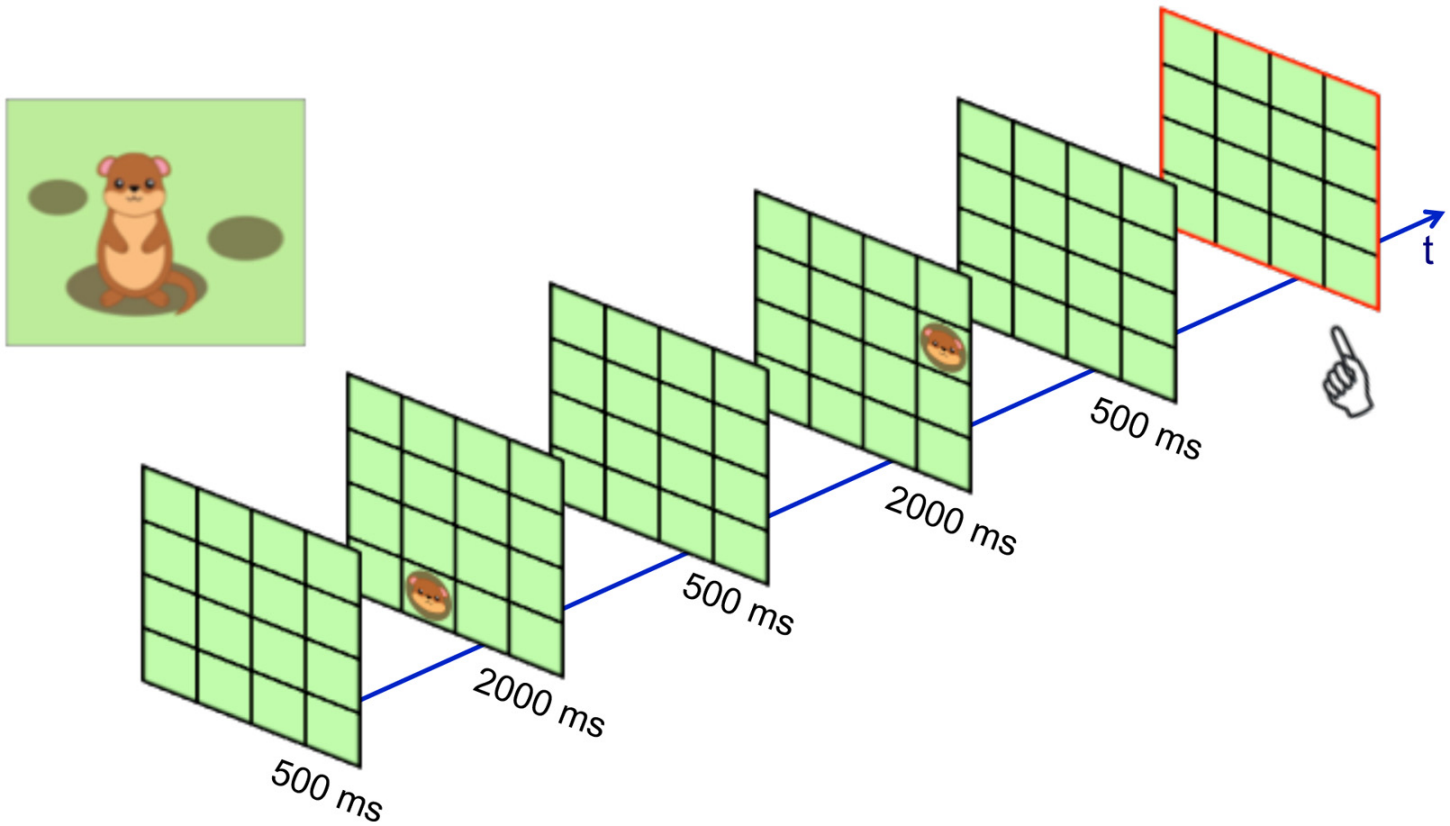
**Example for an item of the starting level (span length of 2) of the Position Span Task**.

Children’s *visuo-spatial working memory* was tested using the *Position Span Task* that was newly designed for the present study. The task was based on the Corsi block-tapping task (Corsi, 1972), but the stimuli were made more child-friendly and presented on a computer monitor (19” laptop, presented with Microsoft PowerPoint). Children saw the head of a groundhog pop up (2 s) in different locations on a green 4 by 4 grid (see **Figure 2**). Between the appearances, the empty grid remained visible (0.5 s). After a sequence of targets, the empty grid showed up with a red frame around it, and children were asked to point to where they had seen the animal pop up in backward order. Span length (i.e., number of targets) started at two and was increased by one after every third trial (up to a maximum of seven targets). If on any difficulty level a child made more than two mistakes, the test was terminated. The task took approximately 5–8 min. Responses were scored with one point for every sequence that was reproduced in the correct order.

Children’s *verbal working memory* was tested using the *Backward Color Recall Task* (Schmid et al., 2008). Children saw a sequence of colored circles (blue, yellow, red, green, brown, black) showing up (1 s) in the center of the white computer screen. Between the appearances, the screen was completely white (1 s). Children were asked to name the colors in backward order when a visual prompt appeared. Span length (number of circles) started at two and was increased by one after every third trial (up to a maximum of seven circles). The test was terminated if a child made two or more mistakes on one difficulty level. The task took approximately 3–5 min. Responses were scored with one point for every sequence that was reproduced in the correct order.

*Verbal IQ* was assessed using the active and passive vocabulary subtests of the HAWIVA-III (Ricken et al., 2007). On the passive vocabulary subtest, children saw four pictures and had to point to the one that the experimenter named; on the active vocabulary test, children were shown one picture and asked to name it. The subtests took about 3–4 min each. Scores were summed across active and passive vocabulary subtests to obtain a general vocabulary score, and transformed into a verbal IQ score according to norm tables.

*Socio-economic Status* was calculated based on parents’ occupations, which were classified according to the ‘International Standard Classification of Occupation’ (ISCO-88, International Labour Office, 1990) and then transformed into an ‘International Socio-Economic Index’ (ISEI: Ganzeboom et al., 1992). We used the higher ISEI of the mother or father. If no present occupation was indicated for either of them, we used the ISEI of the occupation they were trained for. Using this procedure, we were able to determine the SES of all but three children (2%).

## Results

Data of 126 children were available for T1 and T2. In a first step, we scanned each variable for outliers and excluded values that were more than 2.5 standard deviations above or below the mean (1–4 values, 0.8–3% per variable).

The data of the proportional reasoning task were standardized to account for individual response tendencies (cf. Möhring et al., 2015a,b). That is, some children may have used the entire rating scale for their proportional estimations, whereas others may have constrained their responses to only one end of the scale. To account for such individual tendencies to shift responses to one end of the rating scale, we used a within-participant standardization (*ipsatization*; Hicks, 1970). Each child’s individual mean was subtracted from his or her responses and these values were divided by the child’s individual standard deviation. In order to create an index for children’s proportional reasoning performance in terms of their deviation from the normative responses, we calculated the mean absolute difference between these ipsatized responses and the correct (ipsatized) values.

### Descriptives

Means, standard deviations, and ranges of the abilities tested are summarized in **Table 1**. In the balance task, 15 children (5 boys and 10 girls) were able to stand on one leg for the maximum of 100 s. To test for possible sex differences, a MANOVA was calculated with all variables in **Table 1** and SES as dependent variables and sex as a between-participant variable. The analysis showed a significant effect of sex, *F*(9,96) = 1.96, *p* < 0.05, η^2^ = 0.16. *Post hoc* pairwise comparisons (Sidak corrected) revealed that this was mainly due to a significant sex difference in balance skills, with girls showing better performance (*M* = 59.1, *SD* = 31.7) than boys (*M* = 37.6, *SD* = 29.7). There were no further effects of sex (all *p*s > 0.07).

**Table 1:** Means, standard deviations (SD), and ranges of children’s performance in the tasks measuring balance, spatial scaling, and proportional reasoning, as well verbal IQ, inhibition, visuo-spatial and verbal working memory (WM).

		Mean (*SD*)	Range
T1	Balance (in s)	44.88 (31.08)	2–100
	Spatial Scaling (score)	14.91 (2.86)	8–21.33
	Children’s Mental Transformation Task (score)	19.18 (3.27)	10–24
	Verbal IQ	99.84 (10.44)	61–121
	Inhibition (score)	32.26 (8.97)	15.75–57.18
T2	Proportional Reasoning (non-ipsatized deviation in mm)	18.93 (8.33)	7.42–43.67
	Visuo-spatial WM (correct sequences)	6.51 (2.06)	1–11
	Verbal WM (correct sequences)	6.00 (1.70)	2–10

### Correlations

Pearson correlations in **Table 2** show that proportional reasoning, spatial scaling, and mental transformation abilities were significantly related. That is, children with higher spatial scaling and mental transformation scores showed smaller deviations from the normative responses in the proportional reasoning test. More importantly, spatial scaling, mental transformation, and proportional reasoning abilities were also strongly correlated to how long children were able to stand on one leg^2^. Children with better balance skills showed higher scaling and mental transformation scores and smaller deviations in the proportional reasoning task, even after accounting for differences in verbal IQ and sex. Visuo-spatial WM was correlated to balance, spatial scaling, mental transformation, and proportional reasoning (the latter being reduced to a trend when controlled for verbal IQ and sex)^3^. In contrast, verbal WM, inhibitory control, and SES were not significantly correlated to any variables of interest, and were therefore not considered in the following analyses.

**Table 2:** Pearson correlations between balance, spatial scaling, mental transformation (CMTT), proportional reasoning, inhibition, visuo-spatial and verbal WM, as well as the control variables of socio-economic status (SES), verbal IQ, and sex.

	1	2	3	4	5	6	7	8	9	10
(1) Balance	-	0.30^∗∗^	0.29^∗∗^	-0.26^∗∗^	-0.08	0.25^∗∗^	-0.04	0.09	0.08	0.33^∗∗∗^
(2) Spatial Scaling	0.33^∗∗∗^	-	0.38^∗∗∗^	-0.18^∗^	-0.08	0.38^∗∗∗^	0.10	0.17^t^	-0.02	-0.03
(3) CMTT	0.28^∗∗^	0.40^∗∗∗^	-	-0.32^∗∗∗^	-0.08	0.33^∗∗∗^	0.16	0.09	0.28^∗∗^	0.08
(4) Proportional Reasoning^1^	-0.25^∗∗^	-0.20^∗^	-0.25^∗∗^	-	0.02	-0.20^∗^	-0.04	-0.16	-0.34^∗∗∗^	-0.11
(5) Inhibition^1^	-0.06	-0.08	-0.08	0.02	-	-0.07	-0.08	-0.14	-0.01	-0.06
(6) Visuo-spatial WM	0.26^∗∗^	0.38^∗∗∗^	0.32^∗∗∗^	-0.18^t^	-0.07	-	0.17	0.08	0.08	0.03
(7) Verbal WM	-0.02	0.10	0.12	0.01	-0.09	0.16	-	0.04	0.17^t^	-0.09
(8) SES	0.10	0.18^t^	0.05	-0.12	-0.15	0.06	0.00	-	0.18^t^	-0.05
(9) Verbal IQ								-	-	0.15
(10) Sex										-

*Above diagonal: zero-order correlations; below diagonal: partial correlations, controlled for sex and verbal IQ. Missing values were excluded pairwise.*

*^1^Variables with inverse scoring (deviation or interference measures).*

*^∗∗∗^p < 0.001, ^∗∗^p < 0.01, ^∗^ p < 0.05, ^t^p < 0.06.*

### Relation Between Balance Skills and Spatial Scaling

To investigate whether children’s balance skills were related to their ability to scale spatial information even after accounting for effects of control variables and visuo-spatial WM, a hierarchical linear regression analysis was carried out with scaling performance as the predicted variable. As predictor variables, the control variables of sex and verbal IQ were entered in a first step, visuo-spatial WM in a second step, and balance in a third step. Because normal distribution could not be assumed for all residuals, bootstrapped *p*-values are reported here and in the following analyses (Efron and Tibshirani, 1993). Results showed that the control variables and visuo-spatial WM explained 15% of the variance. Visuo-spatial WM was the only significant predictor (β = 0.39, *p* < 0.01). However, even after accounting for these effects, balance still explained an additional, significant part of the variance (Δ*R*^2^ = 0.06, β = 0.27, *p* < 0.01).

### Relation Between Balance Skills and Mental Transformation

To test whether balance skills were connected to children’s mental transformation performance as measured by the CMTT, above and beyond effects of control variables and visuo-spatial WM, we ran a regression analysis similar to the one above, but with the CMTT score as predicted variable. The control variables (sex and verbal IQ) were again entered as predictor variables in a first step, followed by visuo-spatial WM in the second step, and balance in the third step. The analysis revealed that the control variables and visuo-spatial WM accounted for 17% of the variance, with verbal IQ (β = 0.26, *p* < 0.01) and visuo-spatial WM (β = 0.30, *p* < 0.01) being significant predictors. Again, adding balance increased the explained variance significantly (Δ*R*^2^ = 0.04, β = 0.23, *p* < 0.01).

### Relation Between Balance Skills and Proportional Reasoning

To examine whether balance skills were connected to children’s ability to reason about proportions above and beyond effects of control variables and visuo-spatial WM, we ran a regression analysis similar to the one above, but with proportional reasoning as predicted variable. The control variables (sex and verbal IQ) were again entered as predictor variables in a first step, followed by visuo-spatial WM in the second step, and balance in the third step. The analysis revealed that the control variables and visuo-spatial WM accounted for 14% of the variance, with verbal IQ being the strongest and only significant predictor among these variables (β = -0.33, *p* < 0.01). Again, adding balance increased the explained variance significantly (Δ*R*^2^ = 0.04, β = -0.22, *p* < 0.013^4^).

## Discussion

The results of the present study indicate a tight relation between balance skills and spatial scaling as well as mental transformation performance. Hierarchical linear regression analyses revealed that balance explained a significant proportion of the variance in spatial scaling as well as in mental transformation scores, even after accounting for children’s verbal IQ, sex, and visuo-spatial WM. These results extend previous research that investigated the predictive value of balance skills on mental rotation performance (Jansen et al., 2011; Jansen and Kaltner, 2014), and also supports recent research suggesting that spatial scaling is based on similar mechanisms as mental rotation (Möhring et al., 2014).

Regression analyses showed that balance skills in kindergarten explained a significant amount of the variance in proportional reasoning skills at the end of first grade, above and beyond effects of verbal IQ, sex, and visuo-spatial WM. Given that proportional reasoning seems to be closely connected to children’s formal fraction knowledge (Möhring et al., 2015b), it might be considered as an index of formal mathematical skills. Therefore, our results not only indicate a strong relation between motor and spatial abilities, but also point to a connection between motor and some mathematical skills. Future research may clarify how scaling abilities relate to broader and more standardized measures of mathematical abilities or to mathematical abilities that do not rely on proportional reasoning.

Contrary to previous findings (Roebers et al., 2013; Lehmann et al., 2014), motor skill was still a significant predictor for cognitive performance after accounting for effects of WM. The differences to Lehmann et al. (2014) results might be explained by differences in age groups and sample sizes. Lehmann et al. (2014) tested much younger children, who showed bottom effects and very little variance on some variables. Furthermore, the regression analyses of the present study presumably had more power due to a larger sample size and fewer predictors. The discrepancies to Roebers et al. (2013) results could be due to the fact that those results were based on fine-motor skills, whereas the present study focused on a gross-motor skill. Indeed, Piek et al. (2008) showed that gross-motor not fine-motor skills predicted later cognitive performance. But also the cognitive skills that were measured in the two studies differed considerably, with the present study focusing on the much more specific skill of proportional reasoning, rather than academic achievement in general. Future research should take a closer look at the specific roles of fine- and gross-motor skills for academic achievement in general, and math performance in particular.

The present study not only showed that balance was still a significant predictor for spatial as well as proportional reasoning skills after accounting for verbal IQ, SES, and visuo-spatial WM, but also no correlations were found with verbal WM and inhibitory control. These findings rule out the possibility that results might have been driven by individual differences in children’s *general* intelligence or developmental status and, therefore, suggest very *specific* relations between balance skills and spatial and proportional reasoning. The causal direction of these relations cannot be determined based on the present correlational results. However, it is likely that balance skills are foundational for spatial and proportional reasoning abilities, based on findings obtained with cross-lagged designs (Roebers et al., 2013), which showed that motor skills were more predictive for later cognitive performance than vice-versa. Further evidence for a causal role of motor skills comes from experimental training studies showing beneficial effects of infants’ motor experience on cognitive performance (e.g., Needham, 2000; Libertus and Needham, 2010, 2011; Möhring and Frick, 2013; Schwarzer et al., 2013a; Frick and Wang, 2014).

But how may a connection between children’s balance and spatial skills as well as their later proportional reasoning be explained, and why is balance especially such a strong predictor? There are at least two possibilities. On the one hand, balance is an important precondition for many other motor tasks, such as independent locomotion. Thus, having good balance skills may facilitate children’s opportunities to actively explore their spatial environment. This may boost their spatial cognitive skills, as it allows them to build better spatial mental representations of their surroundings and to gain a deeper understanding of spatial relations between objects and agents. On the other hand, good balance skills may be indicative of an effective coordination of visual, proprioceptive, and vestibular information, which might be a precondition for building a stable representation of our spatial environment. For example, in order to perceive the environment as stable, the brain needs to combine single visual inputs (e.g., from fixations between saccades) with information about body posture, head, and eye-movement. Having good balance skills may ensure reliable information from these senses and provide a solid basis for sensory integration, which may be an important precondition for constructing robust spatial representations. As outlined by Gunderson et al. (2012), such robust spatial representations may also play a crucial role in building meaningful spatial representations of numbers, which leads to an improved mathematical understanding (cf. Siegler and Booth, 2004; see also Walsh, 2003, for a theory on how concepts of space and number may be connected).

## Conclusion

Results from the present study showed that balance skills in kindergarten were (a) positively associated with spatial scaling and spatial transformation skills, and (b) predictive for proportional reasoning skills at the end of first grade, even after accounting for children’s verbal IQ, SES, and visuo-spatial WM. Results further suggested that these relations are very specific and not due to general differences in intelligence or executive functions. Future intervention and training studies are needed to clarify the causal role of motor development and the particular importance of balance skills for children’s cognitive development. In our increasingly technological society, and especially in urban environments, children have fewer opportunities to practice gross-motor skills, whereas fine-motor skills may be less restricted. Thus, research on the effects of decreased balance skills on cognitive development is of high relevance, and may have important implications for academic success.

## Author Contributions

AF: conception and design of the study; data acquisition, analysis, and interpretation; drafting parts of and revising manuscript. WM: data analysis and interpretation; drafting parts of and revising manuscript. Both authors approve of the submitted version.

## Conflict of Interest Statement

The authors declare that the research was conducted in the absence of any commercial or financial relationships that could be construed as a potential conflict of interest.

## Footnotes

1 The other four tests were: *Perspective-Taking Test for Children* (PTT-C, Frick et al., 2014), *Ghost Rotation Test* (Frick et al., 2013), *Diagrammatic Representations Test* (DRT, Frick and Newcombe, 2015), and *Cross-sectioning for Children* (Ratliff et al., 2010).

2 These correlations of main interest were still significant if Bonferroni corrections for multiple comparisons were applied. Bonferroni corrections were not applied on the entire correlation matrix in order not to inflate type II error probability, and not to increase the risk of failing to detect possibly important predictors that should be accounted for in the subsequent regression analyses.

3 Normal distributions of the responses could not be assumed for all variables. Therefore, we also ran the correlation analyses using the bootstrapping method (cf. Efron and Tibshirani, 1993). In these analyses the same correlations proved to be significant at *p* < 0.05, except that with bootstrapping, the partial correlation between visuo-spatial WM and proportional reasoning was also significant (*r*_partial_ = -0.20, *p* < 0.05), and the correlation between scaling and proportional reasoning was no longer significant (*r* = -0.14, *p* = 0.15; *r*_partial_ = -0.15, *p* = 0.14).

4 Even with corrected alpha-level of *p* < 0.016 to account for the fact that three regression analyses were performed, all significant predictors remained significant.

## References

[B1] Alloway T. P. Temple K. J. (2007). A comparison of working memory skills and learning in children with developmental coordination disorder and moderate learning difficulties. *Appl. Cogn. Psychol.* 21 473–487. 10.1002/acp.1284

[B2] Archibald S. J. Kerns K. A. (1999). Identification and description of new tests of executive functioning in children. *Child Neuropsychol.* 5 115–129. 10.1076/chin.5.2.115.3167

[B3] Boyer T. W. Levine S. C. (2012). Child proportional scaling: is 1/3=2/6=3/9=4/12? *J. Exp. Child Psychol.* 111 516–533. 10.1016/j.jecp.2011.11.001 22154533

[B4] Bull R. Espy K. A. Wiebe S. A. (2008). Short-term memory, working memory, and executive functioning in preschoolers: longitudinal predictors of mathematical achievement at age 7 years. *Dev. Neuropsychol.* 33 205–228. 10.1080/87565640801982312 PMC2729141 18473197

[B5] Bushnell E. W. Boudreau J. P. (1993). Motor development and the mind: the potential role of motor abilities as a determinant of aspects of perceptual development. *Child Dev.* 64 1005–1021. 8404253

[B6] Campos J. J. Anderson D. I. Barbu-Roth M. A. Hubbard E. M. Hertenstein M. J. Witherington D. (2000). Travel broadens the mind. *Infancy* 1 149–219. 10.1207/S15327078IN0102_1 32680291

[B7] Campos J. J. Bertenthal B. I. Kermoian R. (1992). Early experience and emotional development. *Psychol. Sci.* 3 61–64. 10.1111/j.1467-9280.1992.tb00259.x

[B8] Carlson S. A. Fulton J. E. Lee S. M. Maynard L. M. Brown D. R. Kohl H. W. III (2008). Physical education and academic achievement in elementary school: data from the early childhood longitudinal study. *Am. J. Public Health* 98 721–727. 10.2105/AJPH.2007.117176 18309127 PMC2377002

[B9] Casey M. B. Nuttall R. L. Pezaris E. Benbow C. P. (1995). The influence of spatial ability on gender differences in math college entrance test scores across diverse samples. *Dev. Psychol.* 31 697–705. 10.1037/0012-1649.31.4.697

[B10] Corsi P. M. (1972). Human memory and the medial temporal region of the brain. *Diss. Abstr. Int.* 34:819B.

[B11] Davis E. E. Pitchford N. J. Limback E. (2011). The interrelation of cognitive and motor function in typically developing children aged 4-11 years is underpinned by visual processing and fine manual control. *Br. J. Psychol.* 102 569–584. 10.1111/j.2044-8295.2011.02018.x 21752007

[B12] Diamond A. (2000). Close interrelation of motor development and cognitive development and of the cerebellum and prefrontal cortex. *Child Dev.* 71 44–56. 10.1111/1467-8624.00117 10836557

[B13] Efron B. Tibshirani R. J. (1993). *An Introduction to the Bootstrap.* New York, NY: Chapman & Hall.

[B14] Fischer U. Moeller K. Bientzle M. Cress U. Nuerk H. C. (2011). Sensori-motor spatial training of number magnitude representation. *Psychon. Bull. Rev.* 18 177–183. 10.3758/s13423-010-0031-3 21327351

[B15] Frick A. Hansen M. A. Newcombe N. S. (2013). Development of mental rotation in 3- to 5-year-old children. *Cogn. Dev.* 28 386–399. 10.1016/j.cogdev.2013.06.002

[B16] Frick A. Möhring W. (2013). Mental object rotation and motor development in 8- and 10-month-old infants. *J. Exp. Child Psychol.* 115 708–720. 10.1016/j.jecp.2013.04.001 23708734

[B17] Frick A. Möhring W. Newcombe N. S. (2014). Picturing perspectives: development of perspective-taking abilities in 4- to 8-year-olds. *Front. Psychol.* 5:386 10.3389/fpsyg.2014.00386 PMC4012199 24817860

[B18] Frick A. Newcombe N. S. (2012). Getting the big picture: development of spatial scaling abilities. *Cogn. Dev.* 27 270–282. 10.1016/j.cogdev.2012.05.004

[B19] Frick A. Newcombe N. S. (2015). Young children’s perception of diagrammatic representations. *Spatial Cogn. Comput. Interdiscip. J.* 15 227–245. 10.1080/13875868.2015.1046988

[B20] Frick A. Wang S. (2014). Mental spatial transformations in 14- and 16-month-old infants: effects of action and observational experience. *Child Dev.* 85 278–293. 10.1111/cdev.12116 23647264

[B21] Ganzeboom H. B. G. De Graaf P. M. Treiman D. J. De Leeuw J. (1992). A standard international socio-economic index of occupational status. *Soc. Sci. Res.* 21 1–56. 10.1016/0049-089X(92)90017-B

[B22] Gesell A. Thompson H. A. (1934). *Infant Behaviour: Its Genesis and Growth*. New York, NY: McGraw-Hill.

[B23] Gibson E. J. (1988). Exploratory behavior in the development of perceiving, acting, and the acquiring of knowledge. *Annu. Rev. Psychol.* 39 1–41. 10.1146/annurev.ps.39.020188.000245

[B24] Gunderson E. A. Ramirez G. Beilock S. L. Levine S. C. (2012). The relation between spatial skill and early number knowledge: the role of the linear number line. *Dev. Psychol.* 48 1229–1241. 10.1037/a0027433 22390659 PMC10811729

[B25] Hauf P. (2007). Infants’ perception and production of intentional actions. *Prog. Brain Res.* 164 285–301. 10.1016/S0079-6123(07)64016-3 17920438

[B26] Hicks L. E. (1970). Some properties of ipsative, normative, and forced-choice normative measures. *Psychol. Bull.* 74 167–184. 10.1037/h0029780

[B27] International Labour Office (ed.) (1990). *International Standard Classification of Occupations. ISCO-1988.* Geneva: International Labour Office.

[B28] Jansen P. Heil M. (2010). The relation between motor development and mental rotation ability in 5-to 6-year-old children. *Eur. J. Dev. Sci.* 4 66–74.

[B29] Jansen P. Kaltner S. (2014). Object based and egocentric mental rotation performance in older adults: the importance of gender differences and motor ability. *Neuropsychol. Dev. Cogn. B Aging Neuropsychol. Cogn*. 21 296–316. 10.1080/13825585.2013.805725 23822582

[B30] Jansen P. Schmelter A. Kasten L. Heil M. (2011). Impaired mental rotation performance in overweight children. *Appetite* 56 766–769. 10.1016/j.appet.2011.02.021 21419816

[B31] Knight D. Rizzuto T. (1993). Relations for children in grades 2, 3, and 4 between balance skills and academic achievement. *Percept. Mot. Skills* 76 1296–1298. 10.2466/pms.1993.76.3c.1296 8337082

[B32] Kyttälä M. Aunio P. Lehto J. E. Van Luit J. Hautamaki J. (2003). Visuospatial working memory and early numeracy. *Educ. Child Psychol.* 20 65–76. 10.1136/jnnp-2012-303309

[B33] Kyttälä M. Lehto J. (2008). Some factors underlying mathematical performance: the role of visuospatial working memory and non-verbal intelligence. *Eur. J. Psychol. Educ.* 22 77–94. 10.1007/BF03173141

[B34] LeFevre J.-A. Jimenez Lira C. Sowinski C. Cankaya O. Kamawar D. Skwarchuk S.-L. (2013). Charting the role of the number line in mathematical development. *Front. Psychol.* 4:641 10.3389/fpsyg.2013.00641 PMC3776572 24065943

[B35] Lehmann J. Quaiser-Pohl C. Jansen P. (2014). Correlation of motor skill, mental rotation, and working memory in 3- to 6-year-old children. *Eur. J. Dev. Psychol.* 11 560–573. 10.1080/17405629.2014.888995

[B36] Levine S. C. Huttenlocher J. Taylor A. Langrock A. (1999). Early sex differences in spatial skill. *Dev. Psychol.* 35 940–949. 10.1037/0012-1649.35.4.940 10442863

[B37] Libertus K. Needham A. (2010). Teach to reach: the effects of active vs. passive reaching experiences on action and perception. *Vis. Res.* 50 2750–2757. 10.1016/j.visres.2010.09.001 20828580 PMC2991490

[B38] Libertus K. Needham A. (2011). Reaching experience increases face preference in 3-month-old infants. *Dev. Sci.* 14 1355–1364. 10.1111/j.1467-7687.2011.01084.x 22010895 PMC3888836

[B39] Lopes L. Santos R. Pereira B. Lopes V. P. (2013). Associations between gross motor coordination and academic achievement in elementary school children. *Hum. Mov. Sci.* 32 9–20. 10.1016/j.humov.2012.05.005 23260614

[B40] Luo Z. Jose P. E. Huntsinger C. S. Pigott T. D. (2007). Fine motor skills and mathematics achievement in East Asian American and European American kindergartners and first graders. *Br. J. Dev. Psychol.* 25 595–614. 10.1348/026151007X185329

[B41] MacLeod C. M. (1991). Half a century of research on the Stroop effect: an integrative review. *Psychol. Bull.* 109 163–203. 10.1037/0033-2909.109.2.163 2034749

[B42] Magill F. N. (1996). *International Encyclopedia of Psychology*. London: Fitzroy Dearborn.

[B43] Mix K. S. Cheng Y. L. (2012). The relation between space and math: developmental and educational implications. *Adv. Child Dev. Behav.* 42 197–243. 10.1016/B978-0-12-394388-0.00006-X 22675907

[B44] Möhring W. Frick A. (2013). Touching up mental rotation: effects of manual experience on 6-month-old infants’ mental object rotation. *Child Dev.* 84 1554–1565. 10.1111/cdev.12065 23432700

[B45] Möhring W. Newcombe N. S. Frick A. (2014). Zooming in on spatial scaling: preschool children and adults use mental transformations to scale spaces. *Dev. Psychol.* 50 1614–1619. 10.1037/a0035905 24547996

[B46] Möhring W. Newcombe N. S. Frick A. (2015a). The relation between spatial thinking and proportional reasoning in preschoolers. *J. Exp. Child Psychol.* 132 213–220. 10.1016/j.jecp.2015.01.005 25705050

[B47] Möhring W. Newcombe N. S. Levine S. C. Frick A. (2015b). Spatial proportional reasoning is associated with formal knowledge about fractions. *J. Cogn. Dev.* 10.1080/15248372.2014.996289

[B48] Murray G. K. Veijola J. Moilanen K. Miettunen J. Glahn D. C. Cannon T. D. (2006). Infant motor development is associated with adult cognitive categorisation in a longitudinal birth cohort study. *J. Child Psychol. Psychiatry* 47 25–29. 10.1111/j.1469-7610.2005.01450.x 16405637

[B49] National Research Council (2012). *A Framework for K-12 Science Education: Practices, Crosscutting Concepts, and Core Ideas. Committee on a Conceptual Framework for New K-12 Science Education Standards. Board on Science Education, Division of Behavioral and Social Sciences and Education*. Washington, DC: The National Academies Press.

[B50] Needham A. (2000). Improvements in object exploration skills may facilitate the development of object segregation in early infancy. *J. Cogn. Dev.* 1 131–156. 10.1207/S15327647JCD010201

[B51] Piaget J. (1952). *The Origins of Intelligence in Children (M. Cook, Trans.)*. New York, NY: International Universities Press.

[B52] Piek J. P. Dawson L. Smith L. M. Gasson N. (2008). The role of early fine and gross motor development on later motor and cognitive ability. *Hum. Mov. Sci.* 27 668–681. 10.1016/j.humov.2007.11.002 18242747

[B53] Piek J. P. Dyck M. J. Nieman A. Anderson M. Hay D. Smith L. M. (2004). The relationship between motor coordination, executive functioning and attention in school aged children. *Arch. Clin. Neuropsychol.* 19 1063–1076. 10.1016/j.acn.2003.12.007 15533697

[B54] Planinsec J. (2002). Relations between the motor and cognitive dimensions of preschool girls and boys. *Percept. Mot. Skills* 94 415–423. 10.2466/pms.2002.94.2.415 12027332

[B55] Raghubar K. P. Barnes M. A. Hecht S. A. (2010). Working memory and mathematics: a review of developmental, individual difference, and cognitive approaches. *Learn. Ind. Dif.* 20 110–122. 10.1016/j.lindif.2009.10.005

[B56] Ratliff K. R. McGinnis C. R. Levine S. C. (2010). “The development and assessment of cross-sectioning ability in young children,” in *Proceedings of the 32nd Annual Conference of the Cognitive Science Society*, eds Ohlsson S. Catrambone R. (Austin, TX: Cognitive Science Society), 2816–2821.

[B57] Reuhkala M. (2001). Mathematical skills in ninth-graders: relationship with visuo-spatial abilities and working memory. *Educ. Psychol.* 21 387–399. 10.1080/01443410120090786

[B58] Ricken G. Fritz A. Schuck K. D. Preuss U. (2007). *HAWIVA-III: Hannover-Wechsler-Intelligenztest für das Vorschulalter – III*. Bern: Huber.

[B59] Rigoli D. Piek J. P. Kane R. Oosterlaan J. (2012). An examination of the relationship between motor coordination and executive functions in adolescents. *Dev. Med. Child Neurol.* 54 1025–1031. 10.1111/j.1469-8749.2012.04403.x 22845862

[B60] Roebers C. M. Kauer M. (2009). Motor and cognitive control in a normative sample of 7-year-olds. *Dev. Sci.* 12 175–181. 10.1111/j.1467-7687.2008.00755.x 19120425

[B61] Roebers C. M. Röthlisberger M. Neuenschwander R. Cimeli P. Michel E. Jäger K. (2013). The relation between cognitive and motor performance and their relevance for children’s transition to school: a latent variable approach. *Hum. Mov. Sci.* 33 284–297. 10.1016/j.humov.2013.08.011 24289983

[B62] Röthlisberger M. Neuenschwander R. Michel E. Roebers C. M. (2010). Exekutive funktionen: zugrundeliegende kognitive prozesse und deren korrelate bei kindern im späten vorschulalter. *Z. Entwicklungspsychol. Pädagog. Psychol.* 42 99–110. 10.1026/0049-8637/a000010

[B63] Schmid C. Zoelch C. Roebers C. (2008). Das Arbeitsgedächtnis von 4- bis 5-jährigen kindern: theoretische und empirische analyse seiner funktionen. *Z. Entwicklungspsychol. Pädagog. Psychol.* 40 2–12. 10.1026/0049-8637.40.1.2

[B64] Schwarzer G. Freitag C. Buckel R. Lofruthe A. (2013a). Crawling is associated with mental rotation ability by 9-month-old infants. *Infancy* 18 432–441. 10.1111/j.1532-7078.2012.00132.x

[B65] Schwarzer G. Freitag C. Schum N. (2013b). How crawling and manual object exploration are related to the mental rotation abilities of 9-month-old infants. *Front. Psychol.* 4:97 10.3389/fpsyg.2013.00097 PMC3586719 23459565

[B66] Siegler R. S. Booth J. L. (2004). Development of numerical estimation in young children. *Child Dev.* 75 428–444. 10.1111/j.1467-8624.2004.00684.x 15056197

[B67] Sommerville J. A. Woodward A. L. Needham A. N. (2005). Action experience alters 3-month-old infants’ perception of other’s actions. *Cognition* 96 1–11. 10.1016/j.cognition.2004.07.004 15833301 PMC3908452

[B68] Soska K. C. Adolph K. E. Johnson S. P. (2010). Systems in development: motor skill acquisition facilitates three-dimensional object completion. *Dev. Psychol.* 46 129–138. 10.1037/a0014618 20053012 PMC2805173

[B69] Voelcker-Rehage C. Godde B. Staudinger U. M. (2010). Physical and motor fitness are both related to cognition in old age. *Euro. J. Neurosci.* 31 167–176. 10.1111/j.1460-9568.2009.07014.x 20092563

[B70] Vukovic R. Fuchs L. S. Geary D. C. Jordan N. C. Gersten R. Siegler R. S. (2014). Sources of individual differences in children’s understanding of fractions. *Child Dev.* 85 1461–1476. 10.1111/cdev.12218 24433246 PMC4101068

[B71] Walsh V. (2003). A theory of magnitude: common cortical metrics of time, space, and quantity. *Trends Cogn. Sci.* 7 483–488. 10.1016/j.tics.2003.09.002 14585444

[B72] Woollacott M. Shumway-Cook A. (2002). Attention and the control of posture and gait: a review of an emerging area of research. *Gait Posture* 16 1–14. 10.1016/S0966-6362(01)00156-4 12127181

